# A *Wox3*-patterning module organizes planar growth in grass leaves and ligules

**DOI:** 10.1038/s41477-023-01405-0

**Published:** 2023-05-04

**Authors:** James W. Satterlee, Lukas J. Evans, Brianne R. Conlon, Phillip Conklin, Jesus Martinez-Gomez, Jeffery R. Yen, Hao Wu, Anne W. Sylvester, Chelsea D. Specht, Jie Cheng, Robyn Johnston, Enrico Coen, Michael J. Scanlon

**Affiliations:** 1 School of Integrative Plant Science, Cornell University, Ithaca, NY USA; 2 Marine Biological Laboratory, Woods Hole, MA USA; 3 John Innes Centre, Norwich Research Park, Norwich, UK; 4 State Key Laboratory of Systematic and Evolutionary Botany, Chinese Academy of Sciences, Beijing, China; 5 The Elshire Group Ltd., Palmerston North, New Zealand

**Keywords:** Plant sciences, Genetics, Plant development, Leaf development

## Abstract

Grass leaves develop from a ring of primordial initial cells within the periphery of the shoot apical meristem, a pool of organogenic stem cells that generates all of the organs of the plant shoot. At maturity, the grass leaf is a flattened, strap-like organ comprising a proximal supportive sheath surrounding the stem and a distal photosynthetic blade. The sheath and blade are partitioned by a hinge-like auricle and the ligule, a fringe of epidermally derived tissue that grows from the adaxial (top) leaf surface. Together, the ligule and auricle comprise morphological novelties that are specific to grass leaves. Understanding how the planar outgrowth of grass leaves and their adjoining ligules is genetically controlled can yield insight into their evolutionary origins. Here we use single-cell RNA-sequencing analyses to identify a ‘rim’ cell type present at the margins of maize leaf primordia. Cells in the leaf rim have a distinctive identity and share transcriptional signatures with proliferating ligule cells, suggesting that a shared developmental genetic programme patterns both leaves and ligules. Moreover, we show that rim function is regulated by genetically redundant Wuschel-like homeobox3 (WOX3) transcription factors. Higher-order mutations in maize *Wox3* genes greatly reduce leaf width and disrupt ligule outgrowth and patterning. Together, these findings illustrate the generalizable use of a rim domain during planar growth of maize leaves and ligules, and suggest a parsimonious model for the homology of the grass ligule as a distal extension of the leaf sheath margin.

## Abstract (web summary)

Grass ligules comprise the boundary between the leaf base and the photosynthetic blade; their homology has been debated for over 200 years. Cell-specific gene expression analyses suggest that ligules are homologous to the margins of the leaf base.

## Main

**Fig. 1:**
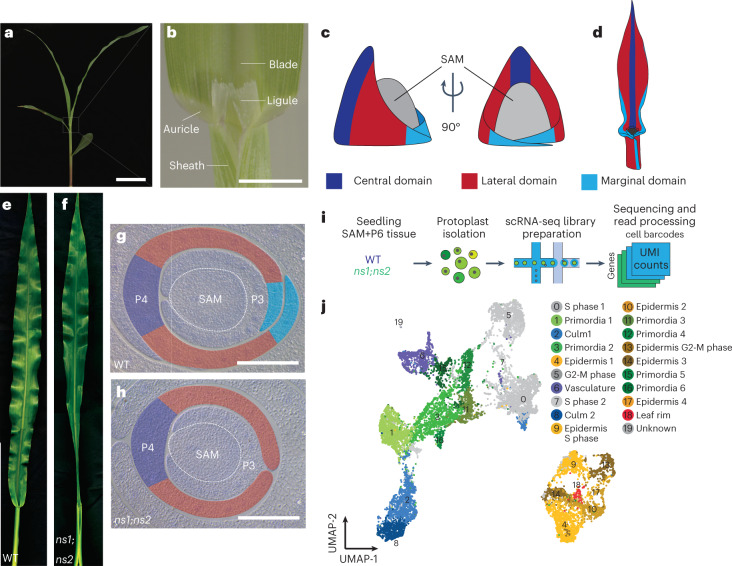
Single-cell transcriptomic analysis of normal and *ns* shoot apices. **a**, The maize seedling shoot. Scale bar, 5 cm. **b**, Morphology of the BSB. Scale bar, 5 mm. **c**,**d**, Illustration of leaf domains in a young leaf primordium (**c**) and a mature leaf (**d**). **e**,**f**, WT (**e**) and *ns* mutant sibling (**f**) showing effect of *ns* mutations on leaf width. **g**,**h**, Transverse sections through WT sibling (**g**) and *ns* mutant (**h**) shoot apices, false coloured to illustrate leaf domains. The tapered marginal domain in WT siblings is deleted in *ns* mutants. Scale bars, 100 μm. **i**, Single-cell transcriptomic approach. **j**, UMAP and embeddings of shoot apex cells coloured by putative cell-type identity. UMI, unique molecular identifier.

The development of the monocot grass leaf has long intrigued plant biologists. Grass leaves are planar, comprising a distal, strap-like blade specialized for photosynthesis and a proximal, sheathing leaf base that encircles and supports the stem (Fig. 1a,b). Leaf development begins from organ initial cells in the peripheral zone of the shoot apical meristem (SAM); these cells comprise at least three mediolateral domains (that is, central, lateral and marginal) that extend bidirectionally from the midvein toward the edges of the leaf (Fig. 1c,d)^1^. The planar, mediolateral outgrowth of both eudicot and monocot leaves is dependent on conserved *Wuschel-like homeobox3* (*WOX3*) transcription-factor-encoding genes^2–4^. As described previously, *Wox3* genes are expressed in the marginal domain of the SAM peripheral zone and at the intersection of the adaxial–abaxial (top–bottom) domains (that is, the rim^1^) of developing leaf primordial edges, where they promote mediolateral outgrowth of the marginal leaf domain^1–4^. In maize, double mutations in the redundant maize *Wox3* homologs *narrow sheath1* (*Ns1*) and *narrow sheath2* (*Ns2*) delete the marginal domain, resulting in narrow leaves that fail to form a sheathing leaf base (Fig. 1e–h)^3–5^. Meanwhile, at the blade–sheath boundary (BSB), grasses form two evolutionarily novel leaf elaborations that are not described in eudicot leaves (Fig. 1b): (1) a hinge-like auricle that angles the blade away from the shoot axis to maximize light capture and (2) the ligule, a fringe of epidermally derived tissue that grows out from the adaxial surface of the leaf and demarcates the BSB^6^. Loss-of-function mutations in the *liguless1* (*Lg1*) gene delete both the ligule and auricle, causing dramatic decreases in leaf angle^6,7^. Natural variation in LG1 activity was a target of selection during domestication to produce the upright leaf angles found in modern cultivated maize^8^. Despite the evolutionary novelty and agricultural importance of the grass ligule and auricle, surprisingly little is known about their morphological homology or developmental mechanisms.

Recent molecular, genetic and computational models of maize leaf development have proposed the existence of a rim domain, defined as cells that promote planar outgrowth at the edges of leaf primordia^1,5^. However, it remains unclear how the rim is genetically specified and whether the rim comprises a distinctive cell type in leaf ontogeny. Moreover, it is uncertain if the ligule also emerges from a rim domain, although transcriptomic analyses of initiating maize ligules indicate that many genes transcribed during leaf initiation are also expressed in ligules^9^. Here, we used a combination of single-cell transcriptomics, higher-order genetics, and molecular–morphogenetic analyses to reveal that a *Wox3*-patterning module underlies rim domain function at the leaf edge, promoting mediolateral outgrowth of the maize leaf and ligule patterning. We propose that this *Wox3*-based rim function is a generalizable mechanism enabling planar outgrowth of plant lateral organs and their elaborations. In addition, our results suggest that the ligule is continuous with the epidermal rim of the leaf sheath, supporting a 150-year-old hypothesis that the ligule develops as a distal extension of the sheath margin^10^.

## Results

### Single-cell transcriptomic analysis reveals a leaf-rim domain

**Fig. 2:**
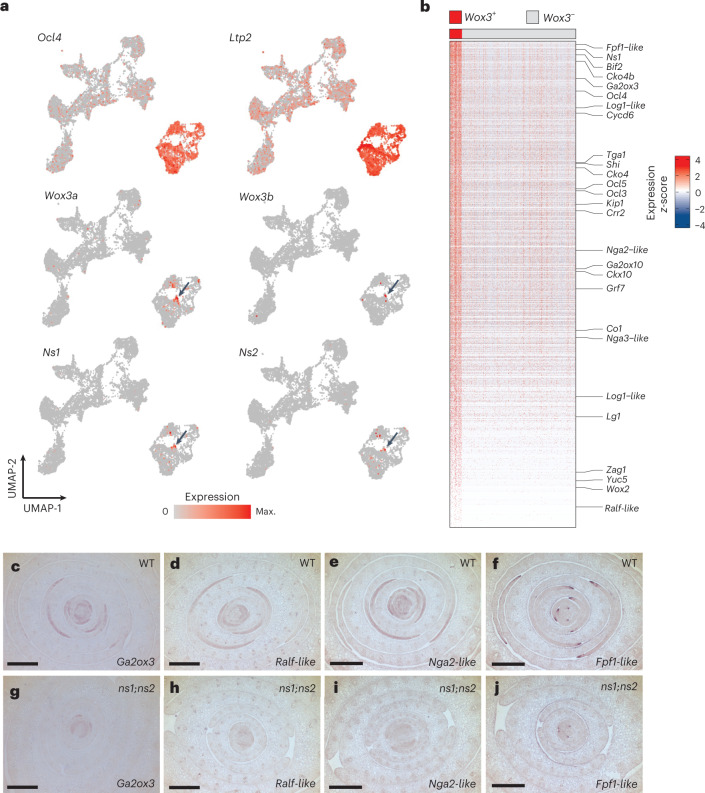
scRNA-seq reveals a leaf primordial rim cell type. **a**, Expression patterns of epidermal marker genes *Ocl4* and *Ltp2* compared with *Wox3* genes. Arrows indicate rim cell cluster. **b**, Heatmap of more than 1,000 gene transcripts upregulated in cells from WT plants expressing one or more *Wox3* genes compared with a randomly selected subset of 1,000 shoot apex cells. Two-sided Wilcoxon rank-sum test, Bonferroni-adjusted *P* < 0.05. **c**–**j**, RNA in situ hybridizations of the rim-upregulated genes *Ga2ox3* (**c**,**g**), *Ralf-like* (**d**,**h**), *Nga2-like* (**e**,**i**), and *Fpf1-like* (**f**,**j**) in WT (**c**–**f**) and *ns* mutant (**g**–**j**) transverse shoot apex sections (*n* = 3). Scale bars, 100 µm. Max., maximum.

**Extended Data Fig. 1:**
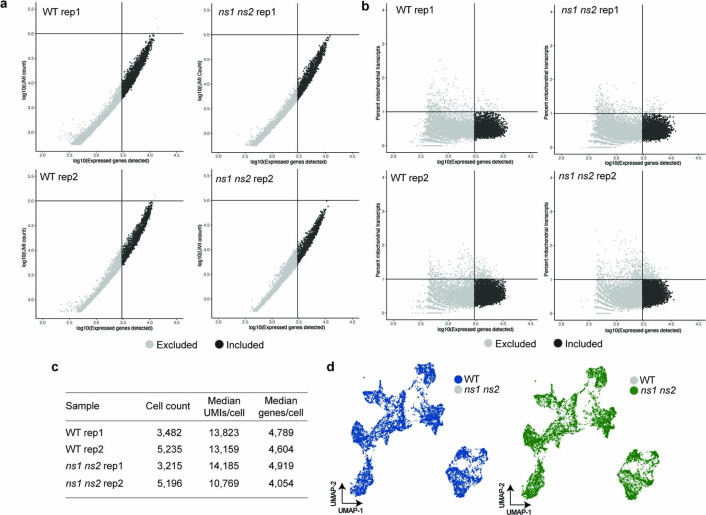
Single-cell transcriptomic cell barcode filtering and summary data. **a**, Filtering of cell barcodes based on minimum genes per cell detected (3,000) and maximum Unique Molecular Identifiers (UMIs, that is transcripts) detected per cell (100,000). **b**, Further filtering of cell barcodes based on percentage of transcripts mapping to mitochondrial genes (1%). **c**, Summary statistics for the four libraries. **d**, UMAP plots with cells labelled by genotype.

**Extended Data Fig. 2:**
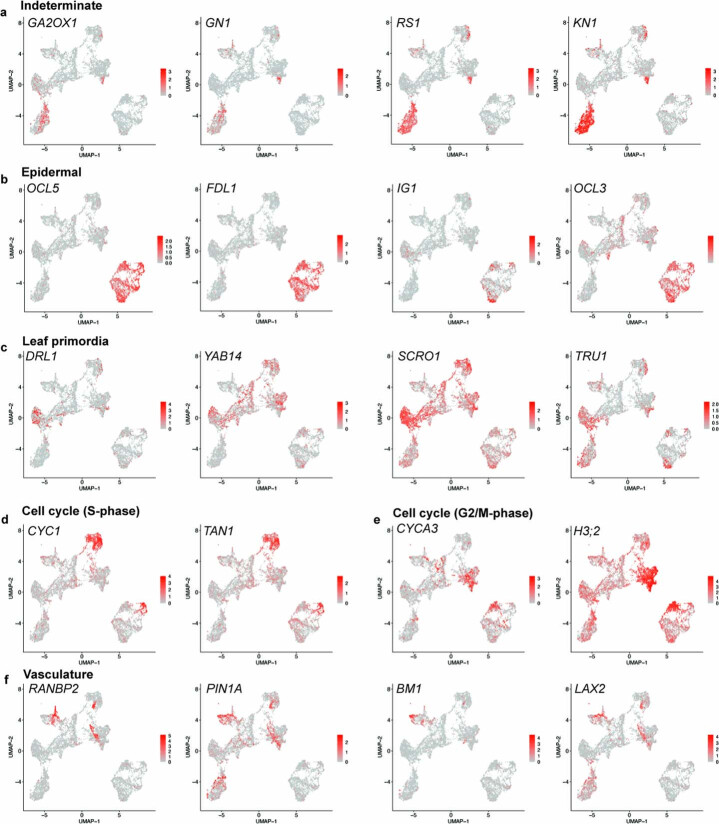
Expression profiles for marker genes used for cell type inference. **a**, Indeterminate/stem markers. **b**, Epidermal marker genes. **c**, Leaf primordia marker genes. **d**, S-phase marker genes. **e**, G2/M-phase marker genes. **f**, Vasculature marker genes. Colour scale indicates UMI (that is transcript) counts per cell.

Models of maize leaf development have invoked a rim domain at the developing leaf primordial edge to direct planar outgrowth^1,5^. We reasoned that the functionally redundant WOX3 family transcription factors NS1 and NS2 (NS1/2) are possible mediators of rim function due to their spatially restricted expression in the marginal rims of leaf primordia and their role in promoting leaf mediolateral outgrowth^4,5^. We used single-cell RNA sequencing (scRNA-seq) to compare the transcriptional profile of cells derived from the shoot apices of phenotypically normal maize seedlings (genotype *Ns1*^*+*^*/ns1; ns2/ns2*, hereafter designated as wild type (WT)) to that of *ns* double mutants expressing the marginal domain deletion phenotype^3^ (genotype *ns1/ns1; ns2/ns2*, hereafter designated as *ns*; Fig. 1i–j). Our aims were (1) to explore the presence of a distinct transcriptional state defining the hypothetical rim domain of leaf primordia and, if detected, (2) to explore whether *Ns1/2* function is required to maintain this transcriptional state and rim function. We performed two replicates of scRNA-seq on dissected shoots containing the shoot apex plus the six youngest leaf primordia (that is, primordium 0 (P0)–P6 (ref. ^6^)) and associated stem tissue. Protoplast samples from WT and *ns* double mutants were processed in parallel using the 10X Genomics Chromium microfluidic system (Fig. 1i). In total, we captured 17,128 cells (8,717 WT cells, 8,411 *ns* cells) and inferred their cell-type identities on the basis of the expression of known cell-type-specific marker genes^11^ (Extended Data Fig. 1a–d and Supplementary Table 1). We identified major cell transcriptional states corresponding to the stem, leaf primordia, vasculature, epidermis and cell cycle across both genotypes (Fig. 1j and Extended Data Fig. 2a–f). Notably, no cluster comprising SAM cells was identified; this failure may be due to the relative scarcity of SAM cells in the sampled tissues and the documented challenges in obtaining plant-shoot stem cells using this microfluidic system^11,12^_._ To search for a rim cell type, we first identified cells expressing the functionally characterized *Wox3* genes *Ns1/2* (ref. ^4^) along with their functionally uncharacterized, duplicate paralogs *Wox3a/b*^13^. Transcripts of all four genes were previously shown to accumulate in the putative rim domain of maize leaf primordia. We found that cells expressing the *Wox3* genes *Ns1/2* and *Wox3a/b* were primarily confined to a single epidermal cell-type cluster (cluster 18) expressing the epidermal marker genes *lipid transfer protein2* (*Ltp2*)^14^ and *outer cell layer4* (*Ocl4*) (Figs. 1j and 2a,b)^15^. Expression was also detected in proliferating S phase and G2-M phase epidermal cells, consistent with the leaf rim being a site of active cell division^5^. Therefore, we considered these *Wox3*-expressing cells to be the most likely candidates for defining the rim cell type.

To explore the transcriptional signatures associated with these putative *Wox3*-expressing rim cells, we performed differential expression analysis. We identified over 1,000 upregulated transcripts (adjusted *P* < 0.05) in the WT *Wox3*-positive cells relative to all other WT cells in our scRNA-seq dataset. We then validated the expression of several of these genes using RNA in situ hybridization, which revealed transcript accumulation at both the leaf edge (that is, putative rim) and in more medial, subepidermal tissue layers of WT leaf primordia (that is, non-rim; Fig. 2b–f and Supplementary Table 2). This suggests that the *Wox3* expression domain is situated at the lateral edge of a broader domain of gene expression that extends medially into the leaf. To determine if these transcriptional patterns are dependent on WOX3 function, we also examined their expression pattern by RNA in situ hybridization in the *ns* double mutant. The expression domains of these marker genes were attenuated in the *ns* mutant (Fig. 2g–j), indicating *Ns1/2* function is required, either directly or indirectly, to maintain accumulation of these transcripts within and medial to the growing leaf edges. Some of these identified differentially expressed genes have previously described functions in growth regulation. For example, *Ga2ox3* regulates cell growth and elongation through gibberellic acid inactivation^16^ and is upregulated in the leaf rim and more medial leaf regions (Fig. 2c,g). Likewise, two *NGATHA2-like* (*Nga2-like*) genes, whose *Arabidopsis* homologs control leaf growth^17^, are upregulated in WT leaf edges and medial regions but show reduced transcript accumulation in *ns* mutants (Fig. 2e,i). Together, these results support the existence of a transcriptionally distinct, WOX3-dependent population of cells at the epidermal leaf edge with the potential to function as a unique rim cell type that is responsible for directing planar outgrowth of the leaf primordium. Hereafter, we refer to this edge-most region of *Wox3* expression as the leaf primordial rim domain.

### Leaf and ligule rims share transcriptional signatures

**Fig. 3:**
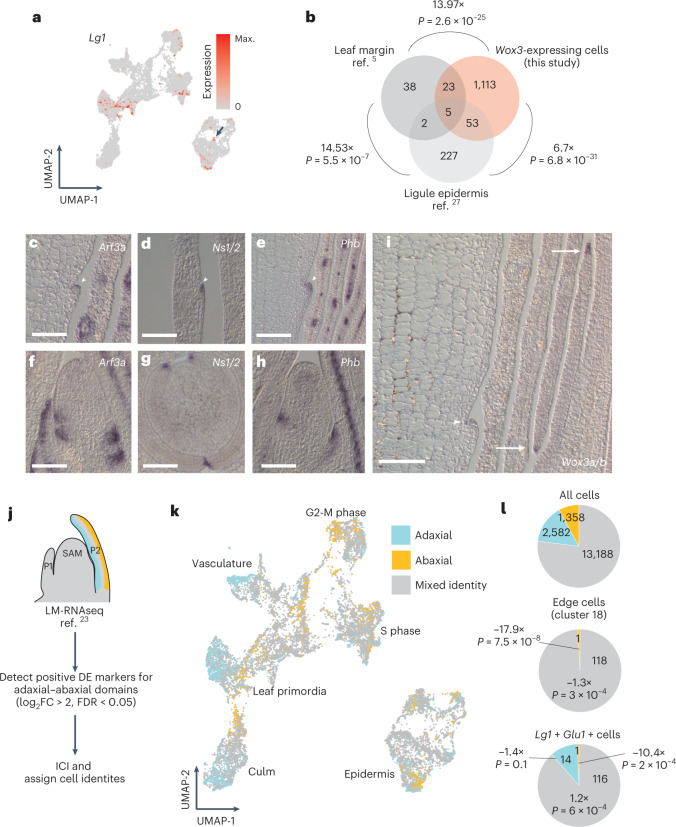
The leaf primordium and its ligule possess rim cells juxtaposed between adaxial–abaxial domains. **a**, Expression pattern of *Lg1*, arrow indicates subset of *Lg1*+ rim cells. Arrow indicates rim cell cluster. **b**, Overlap between upregulated genes in leaf-rim cells and previously collected developing leaf rim and ligule RNA-seq dataset. Numbers indicate fold enrichment and *P* values are the result of a hypergeometric test. **c**–**e**, RNA in situ hybridization of abaxial (*Arf3a*) (**c**), rim (*Ns1/2*) (**d**) and adaxial (*Phb*) (**e**) patterning genes in longitudinal sections of the developing ligule (arrowheads) at the midrib–central domain (**c**,**e**) and marginal domain (**d**). **f**–**h**, Expression of the same indicated patterning genes in median longitudinal sections of the shoot apex (**f**,**h**) and transverse (**g**) sections of the stem. Scale bars, 100 μm. **i**, RNA in situ hybridization of *Wox3a/b* transcripts (arrowhead) in a longitudinal section of the developing ligule at the midrib (central) domain and the leaf rim in the marginal domain (arrows). Scale bar, 100 μm. For micrograph data, *n* = 3. **j**, Scheme for assigning cells to adaxial, abaxial and mixed cell-identity categories. **k**, Colour-coded cell-type identities in the UMAP projection. **l**, Breakdown of cell identities among all cells, rim cells and ligule cells. Cell numbers are in bold, fold enrichment and associated *P* values for subpopulations compared with all cells in the dataset are the output of a two-sided hypergeometric test. FC, fold change; FDR, false discovery rate.

Intriguingly, we observed that the transcription-factor-encoding gene *Lg1* was co-expressed with *Ns1/2* and *Wox3a/b* and appears among the upregulated transcripts within the rim cell type (Fig. 2b). Notably, *Lg1* is also detected in additional cells and tissue layers (Fig. 3a), in agreement with previous reports showing accumulation of *Lg1* transcripts in both epidermal and internal tissue layers during ligule and auricle initiation^9^. *Lg1* is required for ligule and auricle development but is not expressed in the leaf primordial rim^5–7,9^. Moreover, although the marginal domain is deleted in *ns* mutant leaves, the ligule and auricle are otherwise phenotypically normal^3^. The coclustering of cells with ligule and leaf-rim identity suggests that a rim domain may also exist at the initiating ligule. We evaluated the similarity between rim and ligule cell types by comparing transcripts upregulated in rim cells with those upregulated in ligules, as identified in previous laser-microdissection RNA-seq (LM-RNAseq) experiments^5,9^. We found a statistically significant enrichment of shared transcripts in pairwise comparisons of the three datasets (Fig. 3b and Supplementary Table 3). These included genes with growth regulatory functions, such as two cytokinin oxidase enzymes, three *Nga2/3-like* genes, the auxin response regulator *lateral root primordia1* (*Lrp1*)^18^ and the MYB family transcription-factor-encoding gene *fused leaves1 (Fdl1)*, which promotes organ separation^19^. These results suggest that both the leaf and ligule primordia may possess transcriptional signatures of a rim domain that organizes planar growth of both structures.

### Tissue polarity correlates with *Wox3* expression in leaves and ligules

The leaf rim occupies the epidermal edge of developing primordia, at the juxtaposition of adaxial and abaxial leaf domains^1,5^. Ligules, in contrast, arise from the adaxial surfaces of maize leaf primordia^6^. To investigate whether the putative ligule rim also defines an adaxial–abaxial boundary, we asked whether the abaxial and adaxial leaf genes *Arf3a*^20^ and *Phb*^9,21^ are also expressed in ligules (Fig. 3c–h). RNA in situ hybridization revealed that transcripts of both *Arf3a* and *Phb* accumulated in initiating ligules, suggesting both abaxial and adaxial cell identities are present in the adaxially derived ligule. However, the mutually exclusive expression of these two genes, as previously described in leaf primordia^22^, is less well defined in the ligule (Fig. 3c,f,e,h). Transcripts of *Ns1/2* (Fig. 3d) and the paralogs *Wox3a/b* (Fig. 3i) are likewise localized to the epidermally derived distal tip of the growing ligule, at the juxtaposition of adaxial–abaxial gene expression domains, and to the leaf primordia edges (Fig. 3d,i).

**Extended Data Fig. 3:**
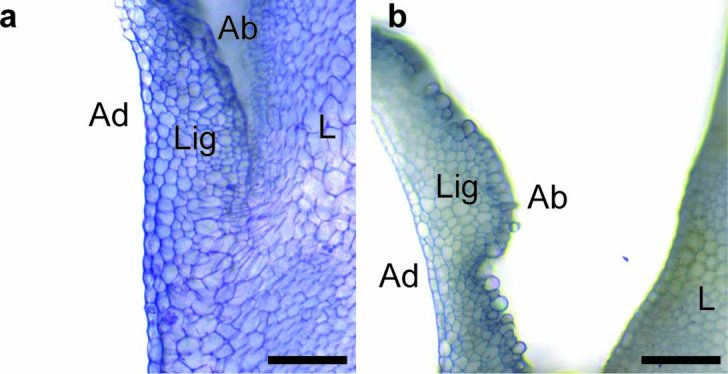
Maize ligules exhibit differences in cell morphology on the adaxial and abaxial epidermal surfaces. **a**, TBO-stained longitudinal hand section of the ligule insertion point on the adaxial leaf surface. **b**, TBO-stained longitudinal hand section of a more distal portion of the ligule. Ad: adaxial ligule surface, Ab: abaxial ligule surface, Lig: ligule, L, leaf. Scale bars: 250 μm, (*n* = 3).

To complement our adaxial–abaxial marker gene expression analyses, we asked whether global transcriptional signatures of adaxial–abaxial identity could be detected using our single-cell transcriptomic data. We leveraged published LM-RNAseq data from the adaxial and abaxial domains of P3 primordia to identify domain-specific marker genes (213 adaxial genes, 93 abaxial genes; log_2_(fold change) > 2, false discovery rate < 0.05)^23^. We then calculated the index of cell identity (ICI) for each cell in our dataset and assessed ICI significance to assign cells to adaxial, abaxial or mixed identities (Fig. 3j,k)^24^. Across all cells in our scRNA-seq data, the majority exhibited a mixed identity, having both adaxial and abaxial character, with a minority of cells having strictly adaxial or abaxial identity (Fig. 3k,l). Among cells in the rim domain population (cluster 18), almost all cells had a mixed identity, consistent with the rim domain being situated at the site of adaxial–abaxial juxtaposition and/or overlap. Consistent with our RNA in situ hybridization results described above (Fig. 3c–i), we found that epidermal cells with initiating ligule identity (that is, expressing ligule markers *Lg1* and *Glu1* (refs. ^9,25^)), exhibited adaxial, abaxial and mixed identities (Fig. 3l). However, adaxial and mixed cell types predominated in the putative ligule-derived cells, probably because the ligule arises from the adaxial surface of maize leaf primordia^6^. Histological analysis of the mature ligule indicates that, similar to leaves, the adaxial and abaxial surfaces of the ligule exhibit distinct epidermal cell morphologies (Extended Data Fig. 3). These data suggest that gene expression signatures of adaxial–abaxial polarity in the initiating ligule (Fig. 3c,e) correspond to asymmetry in the mature organ. Overall, these results support the notion that both leaves and ligules are dorsiventrally asymmetrical and grow via rim function at the juxtaposition of adaxial–abaxial domains.

### *Wox3* mutants perturb the marginal and lateral leaf domains

**Fig. 4:**
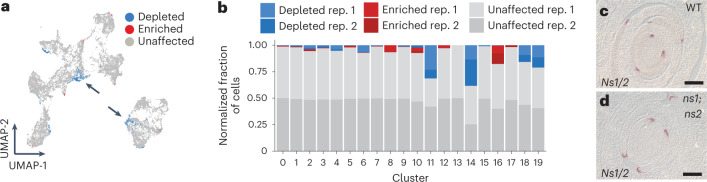
*ns* mutants show a depletion of cell transcriptional states. **a**, Cells with transcriptional profiles identified as more likely to be depleted or enriched in *ns* mutants relative to normal siblings. Arrows indicate *ns*-depleted cells in clusters 11 and 14. **b**, Proportion of depleted and enriched cell states by replicate across clusters. **c**,**d**, RNA in situ hybridization of *Ns1/2* transcripts in transverse sections through the shoot apices of WT (**c**) and *ns* mutant (**d**) seedlings (*n* = 3). Scale bars, 100 μm. Rep., replicate.

If *Ns1/2* are responsible for organizing the rim domain, we predicted that signatures of the rim domain would be perturbed or absent in scRNA-seq data collected from *ns* double mutants in which the marginal leaf domain is deleted^1,3^ (Fig. 1e–h). To determine how cellular transcriptional states were affected by *Ns1/2* mutations, we compared the transcriptomes of cells from *ns* mutant plants to cells from their phenotypically normal siblings. Consistent with the tissue-deletion phenotype of *ns* mutants, perturbation analysis using MELD^26^ identified depletion of epidermal and ground tissue cell types in *ns* mutant samples relative to WT siblings (Fig. 4a,b). However, cells from cluster 18 (that is, cells with rim identity) were only modestly depleted in *ns* mutant seedlings, suggesting the persistence of the rim domain despite the loss of *Ns1/2* function (Fig. 4b). Mutated *ns1;ns2* transcripts also accumulated in *ns* mutant rim cells, despite the fact that both the *ns1* and *ns2* mutant alleles encode non-functional proteins^4^ (Fig. 4c,d). We reasoned the rim cells in *ns* mutant primordia, from which the marginal domain is deleted, may derive from the rims of the intact lateral leaf domain and from ligule domains that persist in *ns* mutant leaves. In addition, the thickened, abnormal leaf edges found in *ns* mutant sheath and blade margins^3^ may contribute to rim cell identity in the *ns* double mutants because they accumulate non-functional *ns1/2* transcripts (Fig. 4c,d). Given the accumulation of *Ns1*/2 paralogous *Wox3a/b* transcripts in the rim, we hypothesized that the genetic basis of rim identity in the *ns* mutant background might be due to *Wox3a/b* function in the lateral domain of the leaf.

**Fig. 5:**
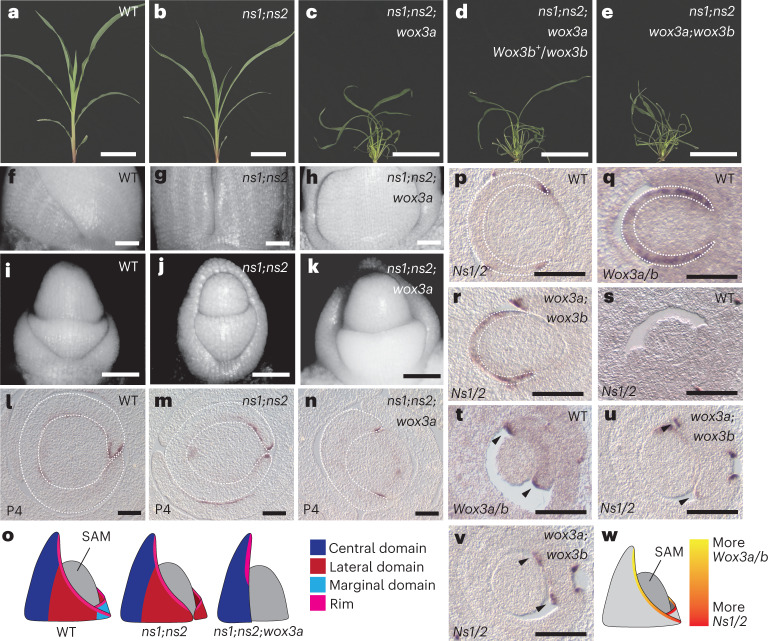
Higher-order *Wox3* mutants perturb mediolateral outgrowth of the leaf primordium. **a**–**e**, Whole shoot phenotypes of WT (**a**), *ns1;ns2* (**b**), *ns1;ns2;wox3a* (**c**), *ns1;ns2;wox3a;Wox3b+/wox3b* (**d**), and *ns1;ns2;wox3a;wox3b* 4-week-old maize seedlings (**e**). Scale bars, 10 cm. **f**–**k**, Epifluorescence microscopy of the P4–P5 disc of insertion (**f**–**h**) and lateral views of P1–P2 (**i**–**k**) in (**f**,**i**) WT, (**g**,**j**) *ns1;ns2*, and (**h**,**k**) *ns1;ns2;wox3a*. **l**–**n**, RNA in situ hybridization of *Cuc2* transcripts in transverse sections through the shoot apices of WT (**l**), *ns* double mutant (**m**) and *ns1;ns2;wox3a* triple mutant (**n**) seedling shoot apices at the P4 stage. White dotted lines outline the P4-staged primordium. Scale bars, 100 μm. **o**, Illustrations of indicated genotypes and their effects on leaf mediolateral domain development. **p**–**r**, RNA in situ hybridization of *Ns1/2* (**p**) and *Wox3a/b* (**q**) in WT and of *Ns1/2* (**r**) in the *wox3a;wox3b* mutant background in transverse sections of early-staged, initiating P1 leaf primordia. Scale bars, 100 μm. **s**,**t**, RNA in situ hybridization of *Ns1/2* (**s**) and *Wox3a/b* (**t**) transcripts in transverse sections through the upper region of WT P2 leaf primordia. Scale bars, 100 μm. **u**,**v**, RNA in situ hybridization of *Ns1/2* transcripts in two serial transverse sections slightly below (**u**) and at the tip (**v**) of the P2 primordium in a *wox3a;wox3b* mutant. Arrowheads indicate expected position of expression foci. Scale bars, 100 μm. **w**, Proposed expression patterns of *Ns1/2* and *Wox3a/b* along the proximodistal axis of the leaf primordial rim. For all micrograph data presented in this figure, *n* = 3.

**Extended Data Fig. 4:**
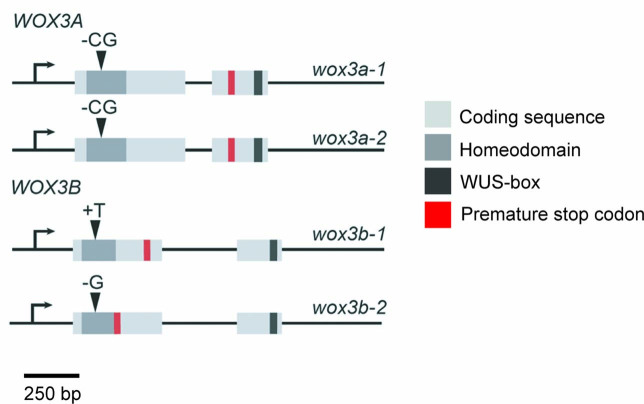
Diagram of *WOX3A/B* CRISPR alleles generated in this study. Arrows indicate the orientation of the transcription start site.

**Extended Data Fig. 5:**
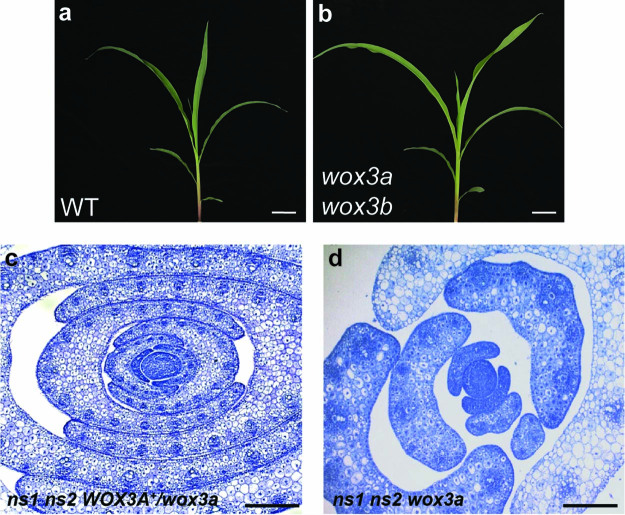
Additional phenotypes of higher-order maize *wox3* mutant plants. (**a-b**) Normal (*NS1*^*+*^*/ns1, ns2*; **a**) and *wox3a wox3b* double mutant plants (**b**) are phenotypically equivalent (*n* = 3). Scale bars = 5 cm. (**c-d**) Effects of *WOX3A* dosage in a *ns1 ns2* background. Transverse cross-sections of the shoot apex of the indicated genotypes stained with toluidine blue. The mediolateral extent of leaves is reduced in *ns1 ns2 WOX3A*^*+*^*/wox3a* mutants (**c**) relative to *ns1 ns2* mutants (compare to Fig. 1h) but less so than in the triple *wox3* mutant (**d**) indicating a dosage effect of *WOX3A* on phenotypic severity (*n* = 3). Scale bars = 250 μm.

To test our hypothesis of *Wox3a/b* function in the lateral rim domain, we used clustered regularly interspaced short palindromic repeats (CRISPR)–Cas9 genome editing to create loss-of-function alleles of the maize *Wox3a* and *Wox3b* genes. Two independently induced mutant alleles were generated for both *Wox3a* and *Wox3b*. Specifically, *wox3a-1* and *wox3a-2* comprise two distinct mutagenic events that each generated an identical CG deletion in the conserved homeobox region of exon 1, which caused a frameshift predicted to induce a premature stop codon in exon 2 of the *Wox3a* transcript; *wox3b-1* contains a T insertion, whereas *wox3b-2* harbours a G deletion in exon 1, both of which are predicted to induce frameshifts and premature stop codons within that exon (Extended Data Fig. 4). Surprisingly, phenotypic characterization of the T_2_ generations revealed that *wox3a;wox3b* double mutants showed no phenotypic differences from their WT siblings (Extended Data Fig. 5a,b), suggesting that some other factor(s) may compensate for loss of *Wox3a/b* function. To test for higher-order functional redundancy between *Ns1/2* and *Wox3a/b*, we generated quadruple mutants of *Ns1/2* and *Wox3a/b* in a segregating F_2_ population. Triple mutant *ns1;ns2;wox3a* plants had much narrower leaves than those observed in WT sibling and in *ns* double mutant plants; triple mutant plants also display severe reductions in plant height and increased leaf curling/twisting (Fig. 5a–e). Notably, plants homozygous for *ns1/2* double mutations and heterozygous for *wox3a* mutations exhibited an intermediate leaf phenotype, which was more severe than *ns* double mutants but less severe than *ns1;ns2;wox3a* triple mutants (Extended Data Fig. 5c–d). Thus, *wox3a* mutations exhibit a dosage-dependent effect on mediolateral leaf outgrowth in the *ns* double mutant background. Moreover, loss of *Wox3b* function had no appreciable effect on the severities of either the *ns* double mutant or the *ns1;ns2;wox3a* triple mutant phenotypes (Fig. 5d,e), indicating *Wox3b* may be dispensable for normal leaf development and does not genetically compensate for loss of paralogous *Wox3* function.

Epiluminescence microscopy of primordia from WT, *ns* double mutant and *wox3* triple mutant seedlings indicates that the reduction of leaf mediolateral outgrowth observed in the *wox3* triple mutant leaf occurs early in development, during the initiation of leaf primordia from the SAM (Fig. 5f–k). Leaf edges of WT P4-staged leaf primordia are overlapped and completely surround the SAM (Fig. 5f,o), whereas equivalently staged *ns* mutant leaf edges fail to overlap due to deletion of the marginal domain, leaving a small gap between the leaf edges^3^ (Fig. 5g,o). Leaf margins of *ns1;ns2;wox3a* triple mutant P4 primordia are widely separated, putatively reflecting failed outgrowth of both the marginal and lateral leaf domains (Fig. 5h,o). Comparative views of WT, *ns* double mutant and *wox3* triple mutant SAM and P1–P2 primordia (Fig. 5i–k) likewise reveal reduced mediolateral growth in proximal regions of *ns* double mutant P2 leaves (Fig. 5j) and more severe mediolateral growth defects in *wox3* triple mutants (Fig. 5k) as compared with phenotypically normal WT siblings (Fig. 5i).

To reveal the mediolateral extent of the leaf base (that is, the proximal-most part of the sheath as it emerges from the node^27^) in the *wox3* mutants, we analysed expression of the developmental boundary gene *Cuc2* via RNA in situ hybridization of transverse shoot sections^1,9^. In WT maize leaves, the sheath fully emerges from the node at the P4 stage of leaf development^28^. At this stage in WT leaf development, *Cuc2* transcripts accumulated only in the margin of the innermost sheath^1^; the outermost sheath edge wraps around the innermost edge to form a sheathing leaf base that encircles the stem^3,27^ (Fig. 5l). In contrast, *Cuc2* accumulation in *ns* double mutant leaf primordia was observed in the edges of both sheaths^1^ (Fig. 5m), which fail to wrap past one another because of the marginal domain deletion^3^. The sheath primordia of the triple mutant *ns1;ns2;wox3a* were markedly reduced in mediolateral development; foci of *Cuc2* expression marked the edges of the mediolaterally stunted primordium (Fig. 5n).

Taken together, these data suggest that, whereas *ns* double mutations delete the marginal domain^1,3^, *wox3* triple mutants also delete the lateral leaf domain. We propose that *Wox3a* and *Ns1/2* redundantly promote development of the lateral domain, whereas only *Ns1/2* are necessary for marginal domain outgrowth from the SAM^3^ (Fig. 5o).

### Compensations in gene expression and *Wox3* redundancy

Although *ns* double mutants delete the marginal domain of maize leaves, mutations in *Wox3a* and *Wox3b* have no effect on maize shoot development (Extended Data Fig. 3). In light of the genetic redundancy revealed by higher-order *wox3* mutants, we tested if differential accumulation of *Wox3* transcripts in leaf domains of initiating P1 primordia might explain the partially redundant *wox3* mutant phenotypes. Specifically, we hypothesized that the localization of *Ns1/2* transcript accumulation may be altered in a compensatory fashion in the rim domains of *wox3a;wox3b* mutants, explaining the lack of a *wox3a;wox3b* double mutant phenotype. As described previously, *Ns1/2* transcripts accumulate in the marginal rim of WT P1 primordia^4,5^ (Fig. 5p). Also consistent with previous reports^13^, *Wox3a/b* transcript accumulation extends from the marginal to the lateral and central rim domains of the WT P1-staged leaf primordium (Fig. 5q). However, in phenotypically normal *wox3a;wox3b* double mutant P1 leaves, *Ns1/2* expression is extended from the marginal to the lateral and central domains (Fig. 5r), a compensatory pattern not observed in WT siblings^4,5^. These data suggest that the absence of a mutant phenotype in *wox3a;wox3b* double mutant leaves is due to compensatory changes in *Ns1/2* expression.

We next examined the relative extent of *Ns1/2* and *Wox3a/b* transcript accumulation in proximodistal domains of maize leaf primordia. Our model of maize leaf mediolateral domains^1^ predicts that the marginal domain encompasses the rim of the entire leaf sheath and extends proximodistally to approximately the midlength of the blade^3^ (Fig. 1d). Our model likewise predicts that the lateral domain comprises the rim of the leaf blade distal to the marginal domain, whereas the central domain occupies the rim at the leaf tip^1^ (Fig. 1d). RNA in situ hybridization of serial-transverse seedling sections revealed that *Wox3a/b* transcript accumulation occurs in the upper, putative lateral-domain rim of WT P2 leaf primordia, whereas *Ns1/2* expression was undetectable in these distal P2 rim domains (Fig. 5s,t). These data indicate that *Wox3a/b* transcripts accumulate higher, in more distal rim domains of the leaf primordium than do *Ns1/2* transcripts. We also observed that, in phenotypically normal *wox3a;wox3b* mutant seedlings, *Ns1/2* expression extended more distally in the P2 primordial rim relative to WT leaves (Fig. 5u,v), suggesting transcriptional compensation in *wox3a;wox3b* double mutant leaf domains occurs proximodistally and mediolaterally (Fig. 5r). We propose that, in WT leaf primordia, *Wox3a/b* and *Ns1/2* expression and function are spatially partitioned, yet overlapping, along the proximodistal axis of the leaf rim (Fig. 5w). Specifically, *Wox3a/b* is expressed in the lateral and marginal domain (Figs. 3i and 5p,s), whereas *Ns1/2* is expressed in the marginal domain (Figs. 3g and 4c).

### Higher-order *Wox3* function and ligule development

**Fig. 6:**
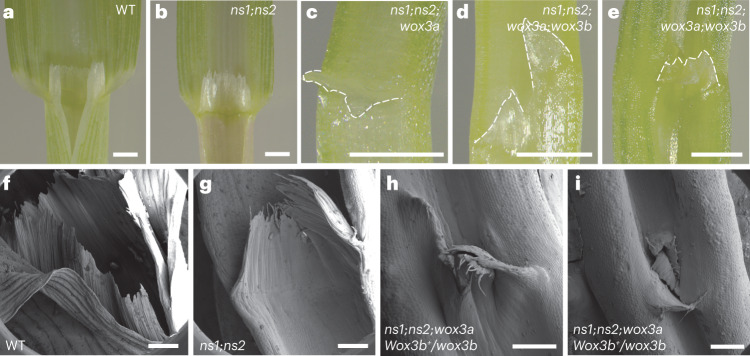
Higher-order *Wox3* mutants perturb ligule development. **a**–**e**, Light micrographs of ligule phenotypes at the BSB of WT (**a**), *ns1;ns2* (**b**), *ns1;ns2;wox3a* (**c**), and *ns1;ns2;wox3a;wox3b* (**d**,**e**) observed by light microscopy. Dotted lines trace distal rim of ligule. Scale bars, 1 mm. **f**–**i**, CryoSEM of the BSB in WT (**f**), *ns1;ns2* (**g**), and *ns1;ns2;wox3a;Wox3b*^*+*^*/wox3b* (**h**,**i**). Scale bars, 500 μm. For all micrograph data presented in this figure, *n* = 3.

**Extended Data Fig. 6:**
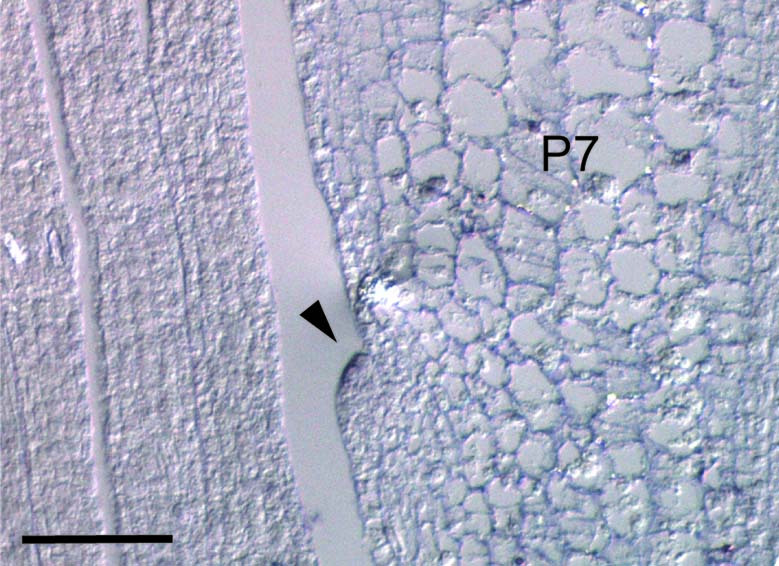
Expression of *NS1/2* in the medial initiating ligule. RNA *in situ* hybridization of a longitudinal histological section a wild type inbred B73 seedling through the midrib-margin plane, showing *NS1/2* expression in the initiating ligule (arrowhead) of the midrib (that is central domain) of the P7-staged leaf primordium. Scale bar, 100 μm.

**Extended Data Fig. 7:**
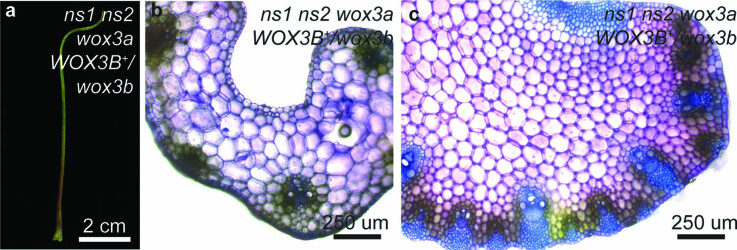
Leaves in *wox3* higher order mutants retain adaxial-abaxial polarity. **a**, A completely liguleless seedling leaf 3 from a triple *wox3* mutant, photographed with the adaxial-side facing in view (*n* = 2). **b**, TBO-stained hand section at the putative blade-sheath boundary shows collinear vascular strands and normal adaxial-abaxial differentiation (*n* = 3). **c**, TBO-stained leaf 3 hand section from a higher order mutant with a ligule also showing retained adaxial-abaxial patterning (*n* = 3).

Our previous LM-RNAseq analyses of ligule initiation detected both *Wox3a/b* and *Ns1/2* transcripts in the ligule lateral domain^9^. Likewise, in situ hybridizations detect *Wox3a/b* and *Ns1/2* expression at the initiating ligule in histological sections of the midrib (that is, central domain; Fig. 3i and Extended Data Fig. 6), and *Ns1/*2 transcripts are detected at the ligule in marginal domains (Fig. 3d). These data suggest that, unlike in leaf primordia, the overlapping expression of *Wox3* paralogs in the ligule primordial rim is extended to include the central, lateral and marginal domains. We next examined whether ligule development was perturbed in *ns1;ns2;wox3a* triple mutants. In WT plants, the ligule forms a long epidermal fringe that spans the width of the mediolateral axis at the BSB^3^ (Fig. 6a,f). Ligule height, localization and continuity are also normal in *ns* double mutants^3^, despite the deletion of the leaf marginal domain and its associated ligule and auricle tissues (Fig. 6b,g). However, numerous ligule developmental defects are observed in *ns1;ns2;wox3a* triple mutants, including displaced, misaligned and disconnected ligule fragments that are reduced in length and fail to span the mediolateral extent of the narrow leaf (Fig. 6c–e,h–i). In juvenile leaves, *wox3* triple mutant ligules are sometimes absent (Extended Data Fig. 7a). As described above for leaf development, *wox3b* mutations had no effect on the phenotypic severity of the ligule defects of *wox3* triple mutants. Notably, *wox3* triple mutant leaves with missing ligules retain adaxial–abaxial patterning at the BSB, indicating that disruption of ligule development is not due to the loss of adaxial–abaxial polarity (Extended Data Fig. 7b,c)^29^.

**Extended Data Fig. 8:**
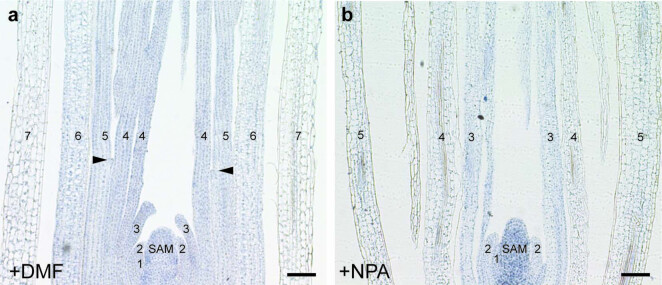
Treatment with the polar auxin transport inhibitor NPA inhibits ligule development. **a**, Lateral section through a vegetative maize inbred line B73 shoot apex cultured on media supplemented with DMF (*n* = 3). **b**, Lateral section through a vegetative inbred line B73 maize shoot apex cultured on media supplemented with NPA dissolved in DMF (*n* = 3). Ligule primordia are absent. Numbers indicated plastochron number. Arrowheads indicate ligule primordia. Scale bars =100 μm.

Because *wox3* triple mutants can form ligules despite the apparent deletions of marginal and lateral leaf domains, we tested whether polar auxin transport is required for ligule initiation, similar to initiating leaf primordia^28^. To this end, maize shoot apices were cultured on control media supplemented with dimethylformamide and on experimental media containing the polar auxin transport inhibitor *N*-1-naphthylphthalamic acid (NPA). Whereas shoot apices cultured in the absence of NPA initiated normal ligules, no ligule primordia were observed in NPA-treated shoot apices (Extended Data Fig. 8). This finding, combined with previous observations of localization of the PIN1-YFP auxin-efflux reporter at the initiating ligule^9,30^, suggests that ligule outgrowth requires auxin transport dynamics. These data support a model wherein development of both leaves and ligules requires polar auxin transport and a redundant WOX3 rim patterning function.

## Discussion

**Fig. 7:**
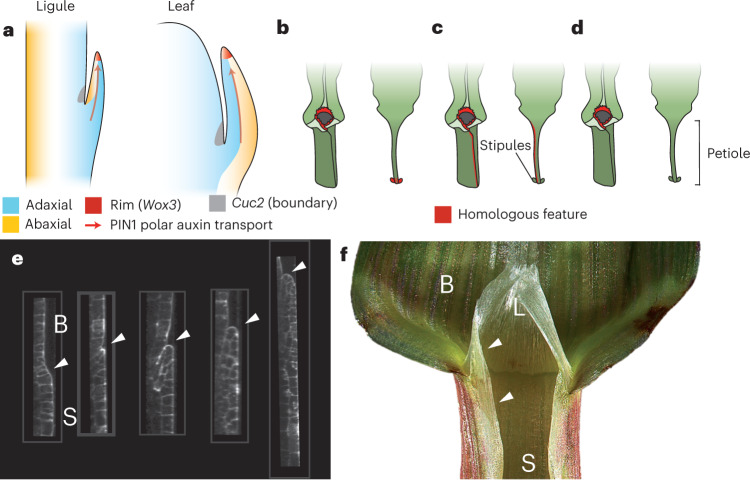
Model for ligule developmental homology. **a**, Model for the elaboration of the grass ligule and the leaf in which WOX3 function in the rim organizes tissue outgrowth between juxtaposed domains of adaxial and abaxial identity. Auxin transport is necessary for both leaf and ligule initiation, and *Cuc2* boundary gene expression is present in both contexts. **b**–**d**, Proposed homologies for the ligule in monocots and eudicots (*Arabidopsis*): grass ligule and sheath margin are homologous to the margins of the eudicot leaf base and stipules (that is, the ligule is a stipule) (**b**), ligule and sheath margins are homologous to the margins of the eudicot petiole (that is, the ligule is an extended sheath margin) (**c**) and the ligule lacks homology with any eudicot leaf feature (**d**). **e**, Confocal fluorescence micrographs of compiled longitudinal sections through ligules at varying stages of maturity (left to right, from leaf). Cells express a pTUBULIN:CFP-TUBULIN construct. The length of the sheath at each stage is indicated (*n* = 3). Arrowheads indicate the ligule cleft. **f**, The BSB of a mature leaf. Arrowheads indicate the continuity between the rim of the sheath and the ligule. B, blade; L, ligule; S, sheath.

**Extended Data Fig. 9:**
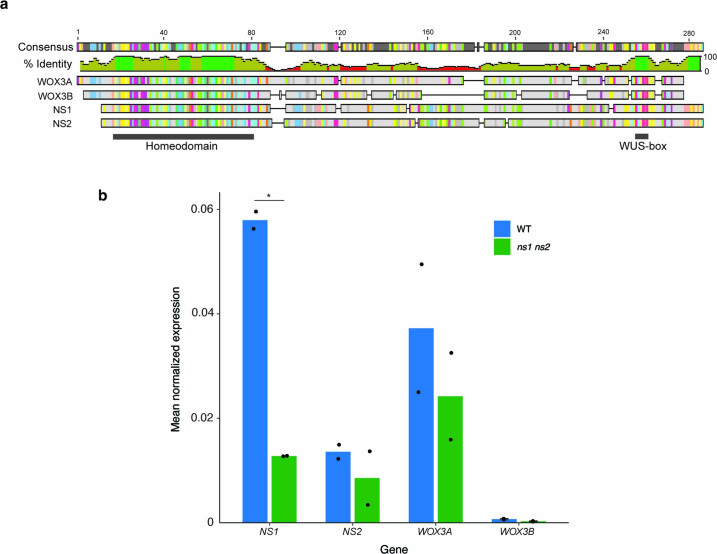
Comparisons of paralogous WOX3 protein sequences and *WOX3* expression levels. **a**, Amino acid alignment of the four maize WOX3 proteins. Amino acids that are conserved in at least two sequences are displayed in colour and protein domains are indicated. Alignment was performed using Geneious alignment software (Genious alignment). **b**, Mean normalized expression of *WOX3* genes in the single-cell RNA-Seq dataset. Statistical significance was determined using a two-tailed Student’s t-test, *P < 0.01. Error bars, s.d. (*n* = 2).

In this study, we identify the transcriptional signatures of a rim cell type expressing redundant WOX3 function at the edges of leaf and ligule primordia. The presence of rim function in developing maize leaves is supported by previous analyses of *ns* double mutants^5^ and was used in computational modelling of both maize and eudicot leaf development^1^. We show that compromised rim function in higher-order *wox3* maize mutants disrupts planar mediolateral outgrowth in leaves and ligules alike (Figs. 5 and 6). Reiterative use and repurposing of genetic patterning modules to sculpt morphogenesis throughout ontogeny is a common theme in development^31^. For example, genes involved in the development of simple leaves in *Arabidopsis* are redeployed during leaflet development in the compound-leaved relative *Cardamine hirsuta*^32^. Moreover, alongside the *Wox3* adaxial–abaxial rim patterning module characterized here, *class I knox*, *Pin* and *Cuc* gene expression are associated with leaf and ligule development in maize^9,33^. These same gene families are also important regulators of leaf and leaflet initiation in eudicots. Similar to initiating leaf primordia, we show that polar transport of the plant hormone auxin is also required for ligule initiation^28^ (Extended Data Fig. 9). Therefore, we suggest that a transport-mediated auxin maximum is required during initiation of both leaf primordia^28,34^ and ligules^9,30^. In this model, auxin precedes the redundant roles of *Wox3* genes in promoting leaf^5,31^ and ligule outgrowth (Fig. 7a). Moreover, ligule and leaves each exhibit adaxial–abaxial juxtapositioning and grow from this RIM domain via WOX3 function (Figs. 3c–h and 6). In this model, the adaxial side of the initiating ligule comprises sheath identity; more experiments are needed to ascertain if the abaxial domain of this emerging ligule comprises sheath, blade or auricle identity.

Open questions remain regarding the functional significance of observed differences in *Wox* gene expression in maize versus *Arabidopsis* leaves. Postinitiation, *Wox3* transcript accumulation quickly converges to the leaf rim in maize, whereas *Arabidopsis WOX*-family leaf genes maintain broader expression in the ‘middle domain’ of the lamina, medial to the leaf edge^35^. Such differences may reflect a role for rim-specific expression of grass *Wox3* genes to reinforce proximodistal growth in the strap-like grass leaf, as compared to *Arabidopsis* leaves. Moreover, compensatory and expanded shifts in expression of *Ns1/2* may explain the lack of a *wox3a/b* mutant phenotype (Fig. 4). It remains unclear why *Wox3a/b* does not compensate for loss of *Ns1/2* function. Promoter and coding sequence-swap experiments may reveal whether changes in transcript accumulation or protein function may be responsible for the partially diverged functions of the maize *Wox3* paralogs.

In addition, *wox3b* mutations have no effects on the severity of *ns1;ns2;wox3a* triple mutant leaf or ligule phenotypes (Figs. 5c–e and 6c–e). We note that the non-mutant *Wox3B* sequence has an intact open reading frame. Comparative alignment of the predicted amino acid sequences of WOX3A and WOX3B reveal high sequence similarity in the homeodomain and the WUS-box domain (Extended Data Fig. 9a), although the functional significance of numerous non-conserved residues outside these regions remains unclear. Perhaps more informatively, our scRNA-seq data reveal that *Wox3b* transcript accumulation is barely detectable in both WT and *ns* mutant seedlings, and is far less abundant than that of *Ns1*, *Ns2*, or *Wox3a* (Extended Data Fig. 9b). We propose that the extremely low level of *Wox3b* expression in maize seedlings may explain why mutations in *wox3b* have no noticeable impact on the severity of the *wox3* triple mutant phenotype.

**Extended Data Fig. 10:**
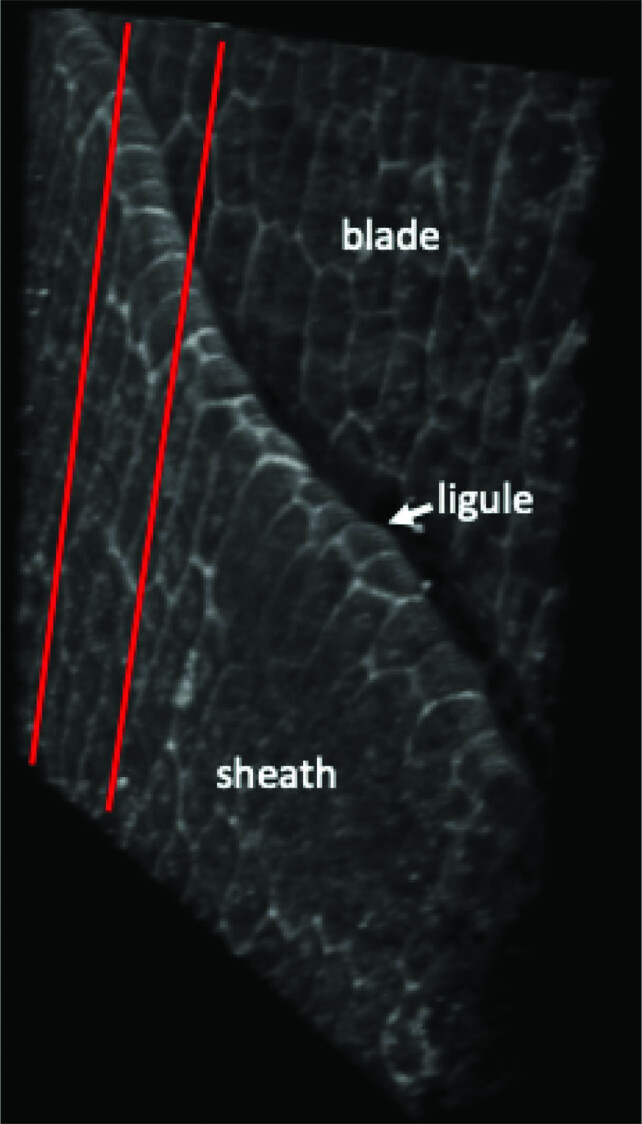
Confocal reconstruction of the developing ligule. Three-dimensional, peridermal, confocal image reconstruction of the surface of one of the same leaf primordia (that is the one with the 5 mm sheath length) shown in the longitudinal image reconstruction presented in Fig. 7e. The red lines delimit the approximate locations where the longitudinal confocal images were collected and compiled for the image in Fig. 7e.

Furthermore, our work sheds light on the evolutionary homology of the grass-specific ligule. For over 150 years, botanists have debated whether the ligule is homologous to the eudicot stipule^36–38^ or comprises a distal extension of the grass sheath margin^10,39^, whereas others have argued that the ligule is a grass-specific evolutionary novelty with no developmentally homologous counterpart in eudicots^40^ (Fig. 7b–d). Classical plant morphologists use explicit criteria to test for organ homology, proposing that homologous organs will occupy equivalent positions in the plant body, share unique derived morphological characters or features and use similar ontogenetic strategies^41^. Recently published work illustrates that the maize sheath is homologous to the eudicot petiole^1^. Thus, early hypotheses that the ligule (located at the distal end of the sheath) is homologous to the stipule (a laterally derived emanation from the eudicot leaf base) violate the criterion of positional homology^41^. Confocal imaging reveals that the initiating ligule orients its growth outward from the distal rim of the sheath; subsequent growth extends the ligule to override the blade (Fig. 7e and Extended Data Fig. 10). Furthermore, we noted that the epidermally derived sheath edges are continuous with the ligule as it extends along the distal rim of the sheath (Figs. 1b and 7f). Our data thus support a hypothesis in which the ligule is an elaboration of the distal end of the sheath edge, and its growth is organized by a leaf-like rim patterning module that comprises marginal, lateral and central leaf domains. Thus, the maize ligule and sheath edges share equivalent positions at the leaf rim and also share special characters and ontogeny, for example, as epidermal elaborations requiring auxin transport and *Wox3* function. In this sense, the ligule and sheath edge satisfy established criteria for homologous structures^41^. Therefore, we propose that the ligule is a derived structure of the grass leaf, homologous to the unelaborated distal margin of the eudicot petiole (Fig. 7c), that is, developmentally patterned by a conserved repertoire of genetic factors.

## Methods

### Genetic stocks and plant growth conditions

Maize seedlings for scRNA-seq and RNA in situ hybridization were grown in 72-well trays in a Percival A100 growth chamber in soil consisting of a 1:1 mixture of Turface MVP and LM111. The day temperature was 29.4 °C, the night temperature was 23.9 °C and the relative humidity was 50%. The day length was 16 h and the relative humidity was 50%. Crosses and phenotypic analysis of CRISPR–Cas9-edited plant material were performed at the Gutermann Greenhouse Facility.

Maize stocks segregating for the narrow sheath mutant phenotype were obtained from the *ns* 1:1 line, proprietary stock generated by a Pioneer Hi-Bred International (Johnston, IA) and donated by M. Albertson as previously described^3^, which has been subsequently introgressed for over 25 generations. Phenotypically WT plants from this line are heterozygous for the *ns1* mutation and homozygous for the *ns2* mutation (genotype *Ns1/ns1-R*, homozygous *ns2-R*), whereas *ns* mutant plants are homozygous for both *ns1-R* and *ns2-R*.

### CRISPR–Cas9 mutagenesis and genotyping

A guide RNA (gRNA) oligonucleotide sequence specifically targeting the homeodomain sequence of the maize *Wox3a* and *Wox3b* genes was designed using CRISPOR. The forward and reverse complement oligonucleotides were synthesized with overhanging sticky ends compatible with cloning into the BsaI site of pRGEB31 (forward sequence TGCTGGATCTGCGACGCGTT). The two complementary oligonucleotides were combined in a 1:1 ratio at a concentration of 2 µM and heated to 95 °C for 5 min, and then allowed to cool to room temperature for 20 min. pRGEBB31 was digested to completion with BsaI and 1 µl of the annealed single gRNA was ligated into 100 ng of pRGEB31 using T4 DNA ligase downstream of the *OsU3* promoter. The ligation product was then transformed into TOP10 competent *Escherichia coli*. Insertion of the gRNA was verified with colony PCR and Sanger sequencing. The gRNA portion of pRGREB31 consisting of the *OsU3* promoter, ligated gRNA and gRNA scaffold, along with the downstream *35* *S promoter*–*Cas9*–*NOS* terminator construct were amplified from pRGEB31 and cloned into a pENTR-dTOPO Gateway Entry vector according to the manufacturer’s instructions (Thermo Fisher Scientific). Sequence verification was done by Sanger sequencing, and a correct clone was introduced into the maize transformation vector pTF101.1 by a Gateway LR clonase reaction. Purified plasmid was supplied to the Iowa State Transformation Facility, where *Agrobacterium*-mediated transformation was used to introduce the pTF101.1 construct harbouring the gRNA and Cas9 constructs into maize inbred B104 callus. Regenerated T_1_ plantlets were transferred to soil. We genotyped T_1_ and T_2_ individuals to identify the nature of the Cas9-generated alleles at *Wox3a* and *Wox3b* using Sanger sequencing of PCR products TA-cloned into pCRII-TOPO vector according to the manufacturer’s instructions. Two independently generated alleles at both loci were carried forward. Individuals carrying alleles that were negative for the Cas9 transgene in the T_2_ generation were used to perform crosses to *ns1;ns2* plants. F_1_ plants from this cross were self-pollinated and ~3,000 resulting F_2_ progeny were screened for phenotypes. Plants were genotyped at the *Wox3a*, *Wox3b*, *Ns1* and *Ns2* loci. DNA was extracted using a guanidium-HCl Whatman paper extraction, as previously described^42^. Genotyping of *Ns1* was performed by PCR amplification with two *Ns1*-specific and a single CACTA-element-specific primer, such that DNA gel electrophoresis banding patterns could be used to detect the presence of the CACTA insertion in the *ns1-R* mutant allele. *Ns2* was genotyped using Sanger sequencing of PCR products to detect the G insertion in the *ns2-R* mutant allele. Primers used for construct design and genotyping are presented in Supplementary Table 4.

### Scanning electron cryomicroscopy

The leaf BSB was dissected and mounted onto a scanning electron microscopy (SEM) stub using conductive graphite adhesive. The sample was lowered into slushed liquid nitrogen, flash frozen and sublimated for 2 min at −70 °C to reduce the formation of ice crystals. The sample was then sputter coated with gold palladium for 30 s at 20 mA before SEM imaging. Samples were imaged on an FEI Strata 400 S DualBeam Focused Ion Beam scanning electron microscope (FIB-SEM) fitted with a Quorum PP3010T scanning electron cryomicroscopy (CryoSEM) or FIB-SEM preparation system.

### RNA in situ hybridization

RNA in situ hybridization probes were prepared as previously described^43^. Primers used for probe cloning are presented in Supplementary Table 4. Images were taken using an Axio Imager Z10 (Carl Zeiss Microscopy) microscope equipped with an AxioCam MRc5 camera.

### Histology

Maize shoot apices were harvested from growth-chamber-grown 2-week-old seedlings and fixed in formaldehyde alcohol acetic acid at 4 °C overnight. Tissues were then dehydrated through a series of 4 °C ethanol solutions (50, 70, 85, 95, 100%), each for 1 h. A 4 °C 100% ethanol incubation was performed overnight, followed by a final room temperature 2 h 100% ethanol incubation the next day. Dehydrated samples were transferred to a 1:1 solution of HistoClear-II (National Diagnostics) for 1 h. A series of three 100% HistoClear-II incubations were then performed, each for 1 h. Samples were placed in a 3:1 mix of HistoClear-II and Paraplast Plus (McCormick Scientific) overnight and heated to 42 °C the next day until the Paraplast dissolved. Samples were then embedded in molten Paraplast over the next 3 days, changing the Paraplast twice daily. Embedded tissues were sectioned using a Leica RM2235 Microtome at a thickness of 10 µm and adhered to Probe-on-Plus microscope slides for RNA in situ hybridization or HistoBond slides for general histological analysis (Thermo Fisher Scientific).

Adhered tissues for toluidine blue (TBO) staining were deparaffinized in HistoClear-II and carried through a decreasing-concentration ethanol series (100% twice, 95, 85, 70, 50, 30%, each for 30 s) and then washed in water. Next, the slides were stained in 0.5% TBO in 1% sodium borate for 2 min and washed twice in water to remove excess TBO. The slides were carried through an increasing-concentration ethanol series (the reverse of the above) and washed twice in HistoClear-II before mounting with Permount.

Hand sections of the BSB were prepared using a razor blade. Tissue was briefly rinsed in de-ionized water and then transferred to 0.5% TBO in 1% sodium borate for 30 s. Samples were rinsed in water to remove excess stain. All samples for histology were imaged using an Axio Imager Z10 (Carl Zeiss Microscopy) microscope equipped with an AxioCam MRc5 camera.

### Confocal microscopy

Confocal microscopy of the developing ligule was performed on maize seedlings expressing pTUBULIN:CFP-TUBULIN (described previously^44^) grown in a growth chamber for 2–3 weeks before live dissection and imaging. At this stage, the BSB of leaf 3 is visually present without dissection, and leaf 4 is emerging with the BSB still inside the sheath bundle. The BSB of leaves 4–7 were dissected out and their sheath lengths were recorded. Samples were laid flat in between a microscope slide and cover slip with the adaxial side up and then imaged on a Zeiss LSM710 Confocal Microscope and camera system at the Cornell University Biotechnology Resource Imaging Center. *Z*-stacks are three-dimensionally reconstructed in Fiji^45^ (ImageJ) software and resliced to obtain longitudinal sections.

### Epiluminescence microscopy

Meristem images were taken using the epi-illumination method^46–48^ as follows. *Zea mays* plants, 2–3 weeks old, were dissected down to 25 mm in length and fixed, under vacuum, in formalin acetic acid alcohol (50% ethanol (EtOH), 37% formalin, 5% glacial acetic acid, 35% H_2_O) for 24 hrs. Samples were dehydrated to 95% EtOH in a serially graded series and then stained with 1% w/v nigrosin solution in 95% EtOH. Samples were further dissected to expose the developing leaves and/or meristem, and image stacks were captured with a Leitz Ultropak incident light illuminator microscope equipped with a Nikon Digital Sights Fi-3 camera running Nikon Elements F software (v.4.60). Focus stacking was performed using the software Picolay (v.2020-10-27) with four filter settings and the ‘2× align’ parameter if alignment was necessary. Images were converted to grayscale, contrast and brightness were adjusted and a scale bar was added in Fiji (v.1.53c).

### Protoplast isolation and scRNA-seq library preparation

Protoplasts were enzymatically isolated from shoot apices as previously described^11^. Briefly, 2-week-old phenotypically mutant *ns1;ns2* and normal *Ns1*^*+*^*/ns1; ns2/ns2* sibling shoots were cut just above the soil. Transverse cuts were made until the diameter of visible stem was approximately 1 mm. Next, a 1-mm-diameter tissue biopsy punch was centred over the stem and used to slice out a cylinder of tissue consisting of the shoot apex and the six to seven most recently initiated leaf primordia. Forceps were used to dissect away excess leaf primordia and the tissue was briefly chopped and added to enzyme solution for 2 hr. Protoplasts were washed and resuspended at a concentration of ~1,000 cells per µl. Protoplasts from each genotype were generated in parallel from ~40 plants per genotype in two replicates.

Protoplasts from each genotype were next loaded into separate wells of a 10X Genomics Chromium single-cell A chip, loading the manufacturer-recommended amount to capture ~10,000 single-cell transcriptomes. Then, 3′ scRNA-seq transcriptomic libraries were prepared according to manufacturer instructions. Complementary DNA and postamplification fragmented libraries were run on a Bioanalyzer to confirm library quality. Separately barcoded libraries from the same replicate were then pooled and sequenced on an Illumina NextSeq 500 sequencer. Library preparation and sequencing were performed at the Cornell University Biotechnology Resource Center.

### scRNA-seq read processing, cell barcode filtering and dimensionality reduction

FASTQ files were generated using the makefastq command in CellRanger v.3.1.0. Next, reads were trimmed, aligned and assigned to cell barcodes using the count command in CellRanger under the default settings. Reads were aligned to the B73 reference genome v3. Because *Wox3b* is unannotated in v3, the genomic sequence of *Ns2* (Zm00001d052598) and its associated annotations from the B73 reference genome v4 were appended to the v3 reference genome. Cell barcodes associated with fewer than 3,000 detected genes, more than 100,000 detected transcripts (that is, unique molecular identifiers) and/or greater than 1% of transcripts derived from mitochondrial genes were excluded from downstream analysis.

The resulting expression count matrices were then merged and analysed in the Seurat R package^49^. scTransform was used for normalization, scaling and log transformation of the count matrix, followed by RunPCA to calculate principal components. The FindNeighbors function was used over dimensions (dims) = 1:25 to construct the shared nearest neighbour graph, followed by FindClusters with resolution of one to assign cells to clusters. The RunUMAP function was used to generate uniform manifold approximation (UMAP) embeddings with the following settings: dims = 1:25, nearest neighbours (n.neighbors) = 25, minimum distance (min.dist) = 0.01 and spread = 1.

Differential expression analysis was performed using the FindMarkers function in Seurat with the Wilcoxon rank-sum test method. Differentially expressed genes were detected by comparing expression in cells expressing at least one *Wox3* gene. To identify markers for each cell-type cluster, the FindAllMarkers function was used with the Wilcoxon rank-sum test method. For all differential expression tests, no minimum fold-change threshold or expression cutoffs were used.

### Sample-associated likelihood analysis

To calculate the likelihood of a cell originating from a particular sample, a sample-associated relative likelihood was calculated for each cell using the MELD algorithm implemented in Python (v.3.8.5)^26^. Briefly, the gene expression count matrix was filtered to include only genes with at least ten transcripts detected over all cells in the dataset. Next, expression levels were normalized on a per-cell basis and square-root transformed. MELD uses graph signal processing to estimate a probability density function for each sample (genotype and replicate) on a graph built over the embedded cell transcriptional space. Three-dimensional UMAP coordinates from Seurat and associated metadata, including genotype and replicate, were therefore provided to MELD. Because optimal graph building parameters need to be determined empirically, the *β* and *k*-nearest neighbour (knn) graph parameters were estimated using the MELD parameter search framework, which permutes cell-sample relationships and calculates the mean squared error (MSE) between sample-associated relative likelihoods for the calculated and ground truth probability density functions. A coarse (knn range of 1–100, step = 5; *β* range of 1–100, step = 5) followed by fine parameter search (knn range of 1–10, step = 1; *β* range of 10–40, step = 1) indicated that parameter values of knn = 8 and *β* = 14 minimized mean squared error and were therefore considered optimal. These values were then used to build the MELD graph and estimate the probability density function for each sample. Normalization of the resulting probabilities on a per-sample basis was then used to calculate the likelihood of a cell deriving from the *ns1;ns2* relative to *Ns1*^*+*^*/ns1; ns2/ns2* sample. A cutoff of 2 s.d. about the mean of the sample-associated likelihood distribution of all cells was then used to determine whether a cell was depleted, unaffected or enriched in the *ns1;ns2* relative to *Ns1*^*+*^*/ns1; ns/ns2* samples. The normalized fraction of cells corresponding to each category was calculated by dividing the number of cells in the category by the total number of cells from that replicate in the cluster.

### Index of cell identity

The ICI was calculated as previously described with some modifications^24,50^. Marker genes for adaxial and abaxial cell fate were determined using previously published RNA-seq data^23^. Reads were downloaded from the Short Reads Archive (SRA: SRP101301) and aligned to the maize B73 genome v3 using STAR (v.2.7.9a)^51^. Reads were counted using HTSeq (v.0.11.2)^52^. Differential expression testing was done using edgeR (v.3.32.1)^53^ to identify differentially expressed genes between the adaxial and abaxial leaf domains. Marker genes were determined as those genes with a log_2_(fold change) > 2 at a Benjamini–Hochberg false discovery rate of 0.05 (Supplementary Table 5). The positive marker genes for the adaxial and abaxial domains determined by edgeR were used to calculate the ICI. A modified formula was used to calculate the gene specificity score *s*_*g*_ for gene *g* in tissue 1 (*t*_1_) relative to tissue 2 (*t*_2_), where CPM is expression in counts per million for a given tissue^47^.

$$s_{g,t_1} = \frac{{{\mathrm{CPM}}_{t_1} - {\mathrm{CPM}}_{t_2}}}{{{\mathrm{CPM}}_{t_1} + {\mathrm{CPM}}_{t_2}}}$$

Following calculation of *s*_*g*_, ICI for each cell was calculated using the following formula:

$${\mathrm{ICI}}_{t_1} = \mathop {\sum}\limits_g^{nt_1} {\frac{{e_g \times s_{g,t_1}}}{{n_{t_1}}} \times \mathop {\sum}\limits_g^{n_{t_1}} {\frac{{{\mathrm{expressed}}(g)}}{{n_{t_1}}}} }$$

Where *e*_*g*_ is the expression level of gene *g* in a given cell, $n_{t_1}$ is the total number of marker genes for tissue 1 expressed in that cell, and expressed*(g)* gives the number of expressed marker genes for tissue 1 in that cell. To determine whether a cell had a significant ICI value for that tissue, a null distribution of ICI scores was created by assigning a random set of genes as tissue marker genes, equivalent in number to the differentially expressed marker genes for each tissue, and the ICI for each tissue calculated for a total of 1,000 permutations. The experimental ICI values for each cell and tissue were then compared with the null distribution of ICI. Experimental ICI values above the fifth percentile of null values were deemed significant and cells were thereby assigned to adaxial, abaxial or mixed cell identity.

### Reporting summary

Further information on research design is available in the Nature Portfolio Reporting Summary linked to this article.

## Supplementary information

Reporting Summary

Supplementary Data 1 Supplementary Tables 1–5. Supplementary Table 1. Differentially expressed genes in each cluster from both *Ns1*^*+*^*/ns1;ns2* and *ns1;ns2* cells. Differential expression was determined by a two-sided Wilcoxon rank-sum test followed by Bonferroni correction. Supplementary Table 2. Markers for *Wox3*-expressing cells in phenotypically normal *Ns1*^*+*^*/ns1;ns2* shoot apices. Differential expression determined by a two-sided Wilcoxon rank-sum test followed by Bonferroni correction. Supplementary Table 3. Overlap of genes upregulated among *Wox3*-positive cells, the developing ligule and the leaf primordium margin. Supplementary Table 4. Oligonucleotides used in this study. Supplementary Table 5. Results of EdgeR two-sided Fisher’s exact test to detect differentially expressed genes between adaxial and abaxial leaf domains. Data from Knauer et al. 2021. A higher log_2_(fold change) indicates higher expression in the adaxial domain.

## Data Availability

RNA-seq data generated in this study are deposited at the National Center for Biotechnology Information Short Reads Archive under BioSample accession code PRJNA924780. Materials used in this study are available upon request.

## Code availability

Analyses of data generated in this study were performed with previously published software as described in the Methods. Code is available at the following GitHub repository: https://github.com/satterleej/WOX3_maize_single_cell.
