# Supplementary material for: A Wox3-patterning module organizes planar growth in grass leaves and ligules
Source: Nat Plants. 2023 May 4;9(5):720–32. doi: 10.1038/s41477-023-01405-0 (PMC10200708; doi:10.1038/s41477-023-01405-0)
Supplement: Supplementary file 1 — Reporting Summary [file 41477_2023_1405_MOESM1_ESM.pdf]

nature portfolio

Corresponding author(s): Michael Scanlon, James Satterlee

Last updated by author(s): Mar 15, 2023

Reporting Summary

Nature Portfolio wishes to improve the reproducibility of the work that we publish. This form provides structure for consistency and transparency in reporting. For further information on Nature Portfolio policies, see our Editorial Policies and the Editorial Policy Checklist.

Statistics

For all statistical analyses, confirm that the following items are present in the figure legend, table legend, main text, or Methods section.

n/a	Confirmed	
☐	☒	The exact sample size (*n*) for each experimental group/condition, given as a discrete number and unit of measurement
☐	☒	A statement on whether measurements were taken from distinct samples or whether the same sample was measured repeatedly
☐	☒	The statistical test(s) used AND whether they are one- or two-sided<br>*Only common tests should be described solely by name; describe more complex techniques in the Methods section.*
☒	☐	A description of all covariates tested
☐	☒	A description of any assumptions or corrections, such as tests of normality and adjustment for multiple comparisons
☐	☒	A full description of the statistical parameters including central tendency (e.g. means) or other basic estimates (e.g. regression coefficient) AND variation (e.g. standard deviation) or associated estimates of uncertainty (e.g. confidence intervals)
☐	☒	For null hypothesis testing, the test statistic (e.g. *F*, *t*, *r*) with confidence intervals, effect sizes, degrees of freedom and *P* value noted<br>*Give P values as exact values whenever suitable.*
☒	☐	For Bayesian analysis, information on the choice of priors and Markov chain Monte Carlo settings
☒	☐	For hierarchical and complex designs, identification of the appropriate level for tests and full reporting of outcomes
☒	☐	Estimates of effect sizes (e.g. Cohen's *d*, Pearson's *r*), indicating how they were calculated

Our web collection on statistics for biologists contains articles on many of the points above.

Software and code

Policy information about availability of computer code

Data collection

No software was used for data collection.

Data analysis

The B73 v3 maize genome and annotations were downloaded from MaizeGDB (https://www.maizegdb.org/)

gRNA design was done using CRISPOR version 5.01 (http://crispor.tefor.net/)

Gene expression analysis was done using the following command line tools:

CellRanger, version 3.1.0
STAR, version 2.7.9a
HTSeq, version 0.11.2

Analysis of single-cell RNA-Seq data and differential expression analysis of bulk expression data were done in R, (version 4.0.2) using the following packages:

Seurat version 4.0.2
edgeR, version 3.32.1

Single-cell perturbation analysis was done in Python (version 3.8.5) using the following software:

MELD version 1.0.0

Custom scripts used for data analysis are available at the following GitHub repository: https://github.com/satterleej/WOX3_maize_single_cell.

WOX3 amino acid sequence alignment was performed with Geneious version 2022.2.1

For manuscripts utilizing custom algorithms or software that are central to the research but not yet described in published literature, software must be made available to editors and reviewers. We strongly encourage code deposition in a community repository (e.g. GitHub). See the Nature Portfolio guidelines for submitting code & software for further information.

Data

Policy information about availability of data

All manuscripts must include a data availability statement. This statement should provide the following information, where applicable:

- Accession codes, unique identifiers, or web links for publicly available datasets
- A description of any restrictions on data availability
- For clinical datasets or third party data, please ensure that the statement adheres to our policy

RNA-Sequencing data generated in this study are deposited at the NCBI Short Reads Archive (SRA) under BioSample accession PRJNA924780. The previously published maize leaf adaxial/abaxial domain laser-capture RNA sequencing data used in this study are available at NCBI Short Reads Archive (SRA) under identifier SRP101301. Laser capture RNA sequencing data from the maize leaf margin and ligule primordia are available in the supplementary materials of the following studies: Conklin et al. 2020 (10.1242/dev.193623) and Johnston et al. 2014 (10.1105/tpc.114.132688). All data and materials used in this study are also available upon request. Cell meta data is available for download at the following GitHub repository: https://github.com/satterleej/WOX3_maize_single_cell.

Human research participants

Policy information about studies involving human research participants and Sex and Gender in Research.

Reporting on sex and gender	Not applicable to this study
Population characteristics	Not applicable to this study
Recruitment	Not applicable to this study
Ethics oversight	Not applicable to this study

Note that full information on the approval of the study protocol must also be provided in the manuscript.

Field-specific reporting

Please select the one below that is the best fit for your research. If you are not sure, read the appropriate sections before making your selection.

☒ Life sciences ☐ Behavioural & social sciences ☐ Ecological, evolutionary & environmental sciences

For a reference copy of the document with all sections, see nature.com/documents/nr-reporting-summary-flat.pdf

Life sciences study design

All studies must disclose on these points even when the disclosure is negative.

Sample size	Sample sizes are described in the paper (methods and figure legends where appropriate). Sample sizes were determined based on experimental feasibility and common practices in the field (see Gala et al. 2021 10.1093/plcell/koab101; Graeff et al. 2021 10.1016/j.molp.2021.07.021; Kim et al. 2021 10.1093/plcell/koaa060 for examples of similar studies).
Data exclusions	No data were excluded.
Replication	All experiments in this study were repeated at least twice with similar results.
Randomization	The different plant genotypes used for experiments in this study were phenotyped/genotyped from randomly segregating population grown in either a greenhouse or growth chamber.
Blinding	Blinding was not applicable to this study. Genotypes of plants used in the analyses in this study were obtained from randomly segregating populations. Samples from each genotype were experimentally observed and analyzed in an equivalent fashion.

Reporting for specific materials, systems and methods

We require information from authors about some types of materials, experimental systems and methods used in many studies. Here, indicate whether each material, system or method listed is relevant to your study. If you are not sure if a list item applies to your research, read the appropriate section before selecting a response.

Materials & experimental systems

n/a	Involved in the study
☒	☐ Antibodies
☒	☐ Eukaryotic cell lines
☒	☐ Palaeontology and archaeology
☒	☐ Animals and other organisms
☒	☐ Clinical data
☒	☐ Dual use research of concern

Methods

n/a	Involved in the study
☒	☐ ChIP-seq
☒	☐ Flow cytometry
☒	☐ MRI-based neuroimaging
